# Genomic Resource of Rice Seed Associated Bacteria

**DOI:** 10.3389/fmicb.2015.01551

**Published:** 2016-01-11

**Authors:** Samriti Midha, Kanika Bansal, Shikha Sharma, Narinder Kumar, Prashant P. Patil, Vasvi Chaudhry, Prabhu B. Patil

**Affiliations:** Bacterial Genomics and Evolution Laboratory, Council of Scientific and Industrial Research-Institute of Microbial TechnologyChandigarh, India

**Keywords:** plant-microbe interactions, diversity, rice seeds, NGS, genomics

Plants are host to diverse microbiome that might have co-evolved since millions of years. This resident microbiota can act as extended genome by contributing in plant growth, development and protection from biotic and abiotic stresses. Rice (*Oryza Sativa*) is a staple food consumed by more than 50% of the world's population. Herein we targeted the bacterial community associated with the healthy rice seeds. In this direction, we isolated and carried out whole genome sequencing of 100 bacterial isolates. These isolates belong to three major bacterial phyla Proteobacteria, Firmicutes, and Actinobacteria that spread over 15 distinct genus and 29 species. A phylogenetic tree based on a broad set of phylogenomic marker genes confirmed the evolutionary relationship amongst the strains and their phylogenetic grouping. Average Nucleotide Identity was also used to establish species identity of isolates that form a particular phylogenetic and taxonomic grouping. The data generated from the present study is one of the first major genomic resources in the field of phytobiome research. Whole genome sequence of the members will be invaluable in this era of big data driven research. Moreover, the majority of genus and species identified in this study are already known for plant probiotic properties. This genomic data with annotation will aid in comparative, evolutionary and ecological studies of bacteria associated with plants or multi-kingdom bacteria associated with nosocomial infections.

## Methods

### Isolation of bacteria from seed

Rice seeds were collected from farmer's field in Fazilka, Punjab, India practicing conventional farming and growing basmati variety. Bacterial isolations were done from a pool of seeds isolated from the same field grown and harvested in three successive years. First isolation was from the seeds harvested in the year 2011, next three isolations were from the crop harvested in 2012 and last from the year 2013. For bacterial extractions, 5 g of seeds were partially crushed (~80%) in normal saline (0.85% NaCl) using sterile pestle and mortar and suspended in 50 ml of the solution (10%; Cottyn et al., 2001). These solutions were incubated for 2 h at 4°C/28°C and then dilution plating was done up to 10^−6^. Samples from each dilution were plated in triplicates on six different media, Peptone Sucrose Agar (PSA), Glucose Yeast extract Calcium carbonate Agar (GYCA), Luria broth (LB) agar, King's B (KB) agar, Nutrient broth (NB) agar, and Potato Dextrose Agar (PDA). Plates were incubated at 28°C and growth was checked up to 6 days. Control plates with/without saline solution were also incubated to check for contamination up to 1 month. Bacterial colonies based on diverse morphology were selected and further processed, as the aim was to capture maximum diversity associated with rice seeds. Bacterial cultures were frozen in 15% glycerol at −80°C.

### Identification by 16S rDNA sequencing

Bacterial isolates were streaked on nutrient broth (NB) agar to get single colonies and 3–4 colonies of each bacterium were suspended into 50 μl of water. Freeze-thaw shock was given to bacteria by freezing the vial at −80°C for 10 min and then incubation at 95°C for 5 min. After that samples were centrifuged at 10,000 rpm for 1 min to collect the supernatant and this step was repeated once again before proceeding further. Samples were quantified for DNA using NanoDrop (Thermo Scientific) and PCR was performed using universal 16S rRNA amplification primers 27F (AGAGTTTGATCMTGGCTCAG) and 1492R (GGTTACCTTGTTACGACTT). After checking for amplification on 1% agarose gel, samples were treated with Exo-Sap (USB, Affymetrix Inc. Cleveland, Ohio, USA) to remove single stranded DNA primers and unused dNTPs and samples were subjected to sanger sequencing using in-house facility ABI DNA sequencer. Data generated in ABI files were visualized in Finch TV v1.4.0 to select the sequences of high quality that were analyzed using Ez-BioCloud (Kim et al., 2012) to identify the closest bacterial species.

From five different isolations, 469 colonies were obtained as a pure culture. Further based on morphological characteristics on the agar plates, the sample size was reduced to 147 for identification of species by 16S rRNA sequencing. Out of these 147 cultures, further shortlisting was done to 100 isolates for genome sequencing, which consisted of minimum one representative of each species from each lot to represent the seed associated bacterial diversity.

### Genome sequencing, assembly, and analysis

Bacterial cultures were revived from −80°C stocks and ZR Fungal/Bacterial DNA isolation kit (Zymo Research) was used to isolate DNA from these. DNA quality check was done using NanoDrop (Thermo Scientific) and agarose gel electrophoresis and quantitation of DNA was performed using Qubit 2.0 Fluorometer (Life Technologies). Sequencing library preparation was performed using Nextera XT sample preparation kit (Illumina, Inc., San Diego, CA, USA) and loaded on to in-house Illumina MiSeq platform (Illumina, Inc., San Diego, CA, USA) using company supplied paired-end sequencing kits. Adapter trimming was done automatically by MiSeq Control Software (MCS) and additional adapter contamination identified by NCBI server was removed by manual trimming. *De novo* assembly of the sequences were done using CLC genomic workbench v7.5 (CLC bio, Aarhus, Denmark) with default settings. Sequences were annotated using NCBI Prokaryotic Genome Annotation Pipeline (http://www.ncbi.nlm.nih.gov/genome/annotation_prok/). RNAmmer 1.2 server was used to annotate the RNA sequences and Ez-BioCloud to identify the closest bacterial species. Protein sequences of 10 known phylogenomic marker genes (*infC, rplB, rplC, rplD, rplE, rplF, rplM, rplN*, and *rplP*) were extracted from the genomic sequences, aligned, and concatenated to obtain multi locus strain phylogeny. These are single copy and universally distributed genes with core housekeeping functions (Wu and Eisen, 2008) and importantly found to be relatively immune to horizontal gene transfer (Jain et al., 1999). Sequences were aligned using Mega v6.0 (Tamura et al., 2013) and a phylogenetic tree was constructed using the Neighbor-Joining method with 500 bootstrap replicates. JSpecies 1.2.1 software was used to calculate Average Nucleotide Identity (ANI) amongst different strains (Richter and Rosselló-Móra, 2009).

### Nucleotide sequences accession numbers

The data has been submitted to NCBI GenBank under accession no. LDPZ00000000-LDTU00000000 and assembly statistics for the 100 bacterial genomes sequenced is provided in Table 1.

**Table 1:** **Assembly statistics and annotation features of bacterial isolates from rice and their accession numbers**.

**S.No**	**Strain**	**Genome size (bp)**	**Coverage(x)**	**Contigs**	**N50(bp)**	**Coding Density (%)**	**Genes**	**tRNA**	**Accession No**.
1	*Aureimonas ureilytica* NS226	5119943	105	287	101071	80.2	4846	58	LDPZ00000000
2	*Aureimonas ureilytica* NS365	5013318	93	183	103973	81.0	4690	56	LDQA00000000
3	*Curtobacterium citreum* NS330	3454135	73	241	29468	81.4	3350	47	LDQB00000000
4	*Curtobacterium luteum* NS184	3586193	100	209	44951	80.2	3460	45	LDQC00000000
5	*Curtobacterium oceanosedimentum* NS263	3370512	97	164	44334	79.7	3206	46	LDRB00000000
6	*Curtobacterium oceanosedimentum* NS359	3421352	94	188	39494	79.4	3258	45	LDRC00000000
7	*Enterobacter asburiae* NS23	4716160	71	102	154724	89.0	4472	71	LDQD00000000
8	*Enterobacter asburiae* NS34	4722850	64	72	165694	88.9	4462	70	LDQE00000000
9	*Enterobacter asburiae* NS7	4728317	166	70	235927	89.0	4464	70	LDQF00000000
10	*Enterobacter cancerogenus* NS104	4884126	117	52	261552	87.7	4630	76	LDQG00000000
11	*Enterobacter cancerogenus* NS111	4874699	99	49	268762	87.7	4622	79	LDQH00000000
12	*Enterobacter cancerogenus* NS188	4889753	117	62	254143	87.8	4644	75	LDQI00000000
13	*Enterobacter cancerogenus* NS31	4869574	96	45	261547	87.8	4620	76	LDQJ00000000
14	*Enterobacter xiangfangensis* NS19	4731803	91	75	347372	88.9	4436	69	LDQK00000000
15	*Enterobacter xiangfangensis* NS24	4705572	98	187	109643	88.7	4444	67	LDQL00000000
16	*Enterobacter xiangfangensis* NS28	4736807	146	38	431385	88.9	4418	73	LDQM00000000
17	*Enterobacter xiangfangensis* NS29	4712167	82	129	160476	88.8	4449	73	LDQN00000000
18	*Enterobacter xiangfangensis* NS371	4725300	125	37	345357	88.8	4402	71	LDQO00000000
19	*Enterobacter xiangfangensis* NS49	4719851	128	33	371391	88.9	4401	70	LDQP00000000
20	*Enterobacter xiangfangensis* NS57	4730229	129	44	265382	88.9	4417	74	LDQQ00000000
21	*Enterobacter xiangfangensis* NS64	4723970	102	76	277091	88.8	4434	71	LDQR00000000
22	*Enterobacter xiangfangensis* NS75	4120285	92	60	131492	88.9	4408	72	LDQS00000000
23	*Enterobacter xiangfangensis* NS80	4722881	85	32	282477	88.9	4405	73	LDQT00000000
24	*Enterobacter xiangfangensis* RSA8	4707136	72	172	134845	88.7	4467	71	LDQU00000000
25	*Exiguobacterium indicum* RSA11	3083121	89	53	109567	88.0	3213	57	LDQV00000000
26	*Exiguobacterium indicum* RSA42	3095093	79	126	69291	87.6	3233	65	LDQW00000000
27	*Kocuria kristinae* RSA28	2302499	205	159	25292	76.4	2072	47	LDRD00000000
28	*Kocuria kristinae* RSA5	2282497	236	204	21282	75.9	2074	47	LDRE00000000
29	*Kocuria kristinae* SA11	2243566	194	202	19601	76.3	2043	44	LDRF00000000
30	*Kocuria kristinae* SA12	2217668	129	249	17427	76.0	2049	46	LDRG00000000
31	*Kocuria kristinae* SA13	2252040	158	233	15889	76.0	2077	44	LDRH00000000
32	*Kocuria kristinae* SA14	2245805	270	175	23072	76.0	1996	43	LDRI00000000
33	*Kocuria kristinae* SA15	2265963	210	211	19130	76.1	2061	47	LDRJ00000000
34	*Leucobacter chromiiresistens* NS354	2841040	101	194	37446	84.6	2618	44	LDRK00000000
35	*Methylobacterium aquaticum* NS228	6323349	110	487	29045	80.0	5861	67	LDRL00000000
36	*Methylobacterium aquaticum* NS229	6377294	110	443	30018	80.1	5907	69	LDRM00000000
37	*Methylobacterium aquaticum* NS230	6353803	127	384	32708	79.9	5835	65	LDRN00000000
38	*Methylobacterium radiotolerans* SB2	6647570	116	334	55436	81.8	6254	47	LDRO00000000
39	*Methylobacterium radiotolerans* SB3	6693841	110	351	55393	81.8	6361	53	LDRP00000000
40	*Microbacterium oxydans* NS234	4013326	114	136	89218	87.8	3976	41	LDRQ00000000
41	*Microbacterium testaceum* NS183	3942620	94	305	37174	84.7	3760	45	LDRR00000000
42	*Microbacterium testaceum* NS206	3881914	113	300	44670	84.7	3687	44	LDRS00000000
43	*Microbacterium testaceum* NS220	4023911	118	291	32198	82.9	3829	43	LDRT00000000
44	*Microbacterium testaceum* NS283	3903457	140	177	50419	86.3	3655	45	LDRU00000000
45	*Microbacterium testaceum* RSA3	3914164	102	222	42959	84.9	3712	45	LDRV00000000
46	*Paenibacillus jamilae* NS115	5586901	49	311	58412	84.5	4997	58	LDRX00000000
47	*Novosphingobium barchaimii* NS277	3553442	135	75	193132	84.3	3332	45	LDRW00000000
48	*Pantoea ananatis* NS296	4734861	69	54	193582	85.2	4439	65	LDQX00000000
49	*Pantoea ananatis* NS303	4733153	80	69	182398	85.3	4421	59	LDQY00000000
50	*Pantoea ananatis* NS311	4721638	73	174	118598	85.1	4449	64	LDQZ00000000
51	*Pantoea ananatis* RSA47	4738039	91	46	229877	85.2	4439	65	LDRA00000000
52	*Pantoea dispersa* NS215	4843865	84	35	271354	86.9	4425	67	LDRY00000000
53	*Pantoea dispersa* NS375	4887220	81	42	318516	86.5	4510	73	LDRZ00000000
54	*Pantoea dispersa* NS380	4801064	74	123	195710	86.3	4454	70	LDSA00000000
55	*Pantoea dispersa* NS389	4819785	73	54	228728	86.5	4420	61	LDSB00000000
56	*Pantoea dispersa* RSA31	4949525	90	194	266158	86.7	4607	64	LDSC00000000
57	*Pantoea dispersa* SA2	4906145	52	52	255271	85.8	4488	66	LDSD00000000
58	*Pantoea dispersa* SA3	4905368	72	83	185303	85.9	4494	65	LDSE00000000
59	*Pantoea dispersa* SA4	4911078	55	58	156533	85.8	4503	68	LDSF00000000
60	*Pantoea dispersa* SA5	4897186	77	98	190714	85.9	4500	64	LDSG00000000
61	*Pantoea stewartii* NS381	4693026	82	51	268280	84.7	4338	68	LDSH00000000
62	*Pantoea stewartii* RSA13	4766695	96	48	167594	84.4	4423	67	LDSI00000000
63	*Pantoea stewartii* RSA30	4760407	65	84	211952	84.3	4420	65	LDSJ00000000
64	*Pantoea stewartii* RSA36	4791295	139	76	285765	84.2	4451	64	LDSK00000000
65	*Pseudacidovorax intermedius* NS331	5547045	97	295	34362	84.5	5076	49	LDSL00000000
66	*Pseudomonas parafulva* NS212	4823858	147	147	107693	86.7	4359	65	LDSM00000000
67	*Pseudomonas parafulva* NS96	4672265	177	162	108451	87.2	4283	64	LDSN00000000
68	*Pseudomonas psychrotolerans* NS2	5271410	146	130	143537	86.4	4687	59	LDSO00000000
69	*Pseudomonas psychrotolerans* NS201	5262847	139	172	119605	86.7	4783	55	LDSP00000000
70	*Pseudomonas psychrotolerans* NS274	5418049	135	103	95239	86.2	4848	60	LDSQ00000000
71	*Pseudomonas psychrotolerans* NS337	4837497	62	192	118956	88.0	4559	59	LDSR00000000
72	*Pseudomonas psychrotolerans* NS376	5326810	95	136	99843	86.0	4790	60	LDSS00000000
73	*Pseudomonas psychrotolerans* NS383	5413173	113	174	94742	86.0	4877	55	LDST00000000
74	*Pseudomonas psychrotolerans* RSA46	5455836	120	197	97624	86.0	4934	59	LDSU00000000
75	*Pseudomonas psychrotolerans* SB11	5202479	113	232	134485	86.7	4817	59	LDSV00000000
76	*Pseudomonas psychrotolerans* SB14	5345440	124	169	129021	86.0	4823	61	LDSW00000000
77	*Pseudomonas psychrotolerans* SB18	5339762	147	173	104148	86.4	4889	59	LDSX00000000
78	*Pseudomonas psychrotolerans* SB5	5435284	113	171	92521	86.1	4884	60	LDSY00000000
79	*Pseudomonas psychrotolerans* SB8	5130436	138	116	137111	86.5	4598	60	LDSZ00000000
80	*Pseudomonas psychrotolerans* SB9	5283903	104	121	91801	86.7	4803	57	LDTA00000000
81	*Sphingomonas endophytica* NS334	3642958	164	330	58696	85.2	3506	47	LDTB00000000
82	*Sphingomonas sanguinis* NS258	3868819	129	346	36442	85.0	3708	45	LDTC00000000
83	*Sphingomonas sanguinis* NS319	4169594	110	280	33184	84.9	3912	48	LDTD00000000
84	*Sphingomonas sanguinis* SB4	4019921	97	226	40305	84.5	3772	49	LDTE00000000
85	*Sphingomonas yabuuchiae* NS355	3910679	133	190	45578	85.0	3612	49	LDTF00000000
86	*Staphylococcus epidermidis* SA6	2540147	111	66	227679	82.1	2443	62	LDTG00000000
87	*Staphylococcus epidermidis* SA8	2505031	84	181	30972	82.4	2419	49	LDTH00000000
88	*Staphylococcus epidermidis* SB7b	2506997	86	148	44745	82.5	2423	55	LDTI00000000
89	*Staphylococcus epidermidis* SB7c	2559409	118	96	109441	82.3	2485	58	LDTJ00000000
90	*Staphylococcus sciuri* NS1	2918429	80	127	70746	85.4	2972	54	LDTK00000000
91	*Staphylococcus sciuri* NS112	2878717	73	136	49833	85.3	2926	52	LDTL00000000
92	*Staphylococcus sciuri* NS202	2743697	84	56	93562	86.8	2757	56	LDTM00000000
93	*Staphylococcus sciuri* NS36	2881364	90	117	52335	85.3	2907	54	LDTN00000000
94	*Staphylococcus sciuri* NS44	2979824	160	164	142946	84.5	3038	55	LDTO00000000
95	*Staphylococcus sciuri* NS53	2897114	85	155	53334	85.3	2951	51	LDTP00000000
96	*Staphylococcus sciuri* RSA37	2890453	130	72	81742	85.5	2911	54	LDTQ00000000
97	*Staphylococcus warneri* NS346	2563869	96	87	84728	83.6	2485	50	LDTR00000000
98	*Staphylococcus warneri* SA10	2549551	84	145	36722	82.4	2497	53	LDTS00000000
99	*Staphylococcus warneri* SA9	2541660	96	77	114371	82.0	2482	56	LDTT00000000
100	*Staphylococcus xylosus* NS341	2842280	89	111	59876	82.2	2702	54	LDTU00000000

### Interpretation of data set

**Figure 1:**
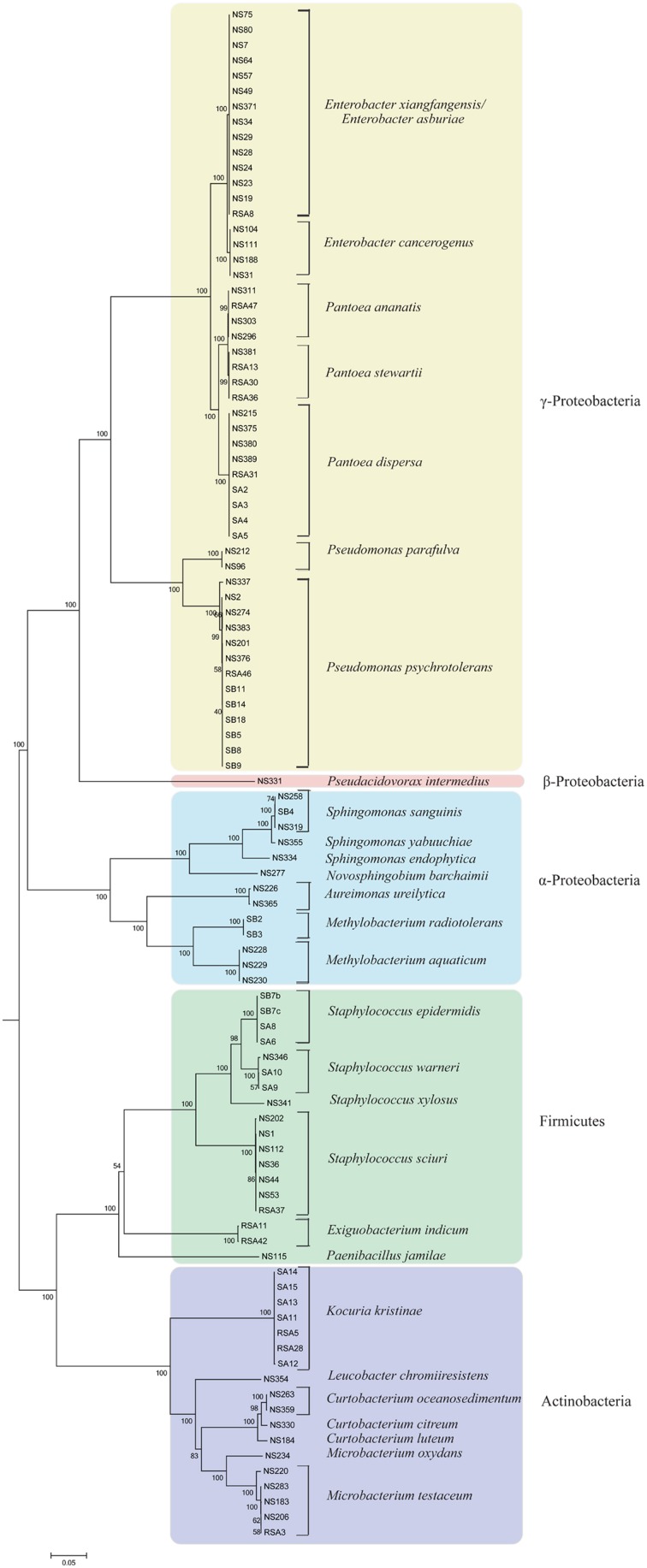
**Multi locus sequence analysis of rice seed associated bacterial isolates constructed using 10 phylogenomic marker genes with Neighbor-Joining method and 500 bootstrap replications**. Bacterial strains belonging to different phylum; Proteobacteria (α, β, and γ), Firmicutes, and Actinobacteria, are highlighted with different background colors. Strains belonging to each species are grouped together with high boot strap values.

High-quality sequencing data generated for each strain (Table 1) was *de novo* assembled with coverage ranging from 49x to 270x. Analysis based on complete 16S rRNA sequence extracted from the whole genome sequences assigned them to 15 distinct genus and 29 species. It is also pertinent to note that genus/species assignment has been validated by a new QA protocol of NCBI during submission process. Here, the “input fasta sequences are BLASTed against a collection of 23 bacterial ribosomal protein COG families during submission.” Multilocus phylogenetic tree based on marker genes further supports the distinction between different groups of bacteria (Figure 1) to strain level. Description of the strains assigned to 15 different genera is provided below:

#### Genus: *Kocuria*

*Kocuria* is a gram-positive bacterium, belonging to phylum Actinobacteria. Seven isolates (SA11, SA12, SA13, SA14, SA15, RSA5, and RSA28) belonging to this genus were sequenced from two different libraries and two different year lots. Complete 16S rRNA typing has assigned the seven isolates to same species *Kocuria kristinae.* Further ANI analysis showed the seven strains have genome level identity >99.8%, much above the cut-off of 94–96% for delineation of species (Konstantinidis and Tiedje, 2005; Richter and Rosselló-Móra, 2009) and suggests their monophyletic/clonal nature. Interestingly only in case of *Kocuria*, a single species was detected even after having multiple strains.

#### Genus: *Curtobacterium*

*Curtobacterium* is also a gram-positive bacterium belonging to phylum Actinobacteria and class Microbacteriaceae. Four isolates of *Curtobacterium* were obtained from seed microbiome that belong to three different species on the basis of 16S rRNA sequences i.e. *Curtobacterium luteum* (NS184), *Curtobacterium citreum* (NS330), and *Curtobacterium oceanosedimentum* (NS263, NS359), sequenced from two different libraries. ANI values amongst the genomes of four strains also support the presence of three species.

#### Genus: *Leucobacter*

*Leucobacter* is another gram-positive Actinobacteria, belonging to class Microbacteriaceae. One isolate NS354 belonging to this genus was isolated from rice seeds and was assigned to *Leucobacter chromiiresistens* on the basis of 16S rRNA sequences.

#### Genus: *Microbacterium*

*Microbacterium* is another gram-positive Actinobacteria and six isolates from rice seeds were assigned to genus *Microbacterium*. These isolates were extracted from three different libraries and two different rice lots. 16S rRNA sequences assigned them into two different species *Microbacterium testaceum* (NS183, NS206, NS220, NS283, and RSA3) and *Microbacterium oxydans* (NS234), while ANI values suggest NS220 to be a different species as the values are less than 87.5% with all the other strains.

#### Genus: *Exiguobacterium*

*Exiguobacterium* is a gram-positive bacterium that is assigned to phylum Firmicutes. Two isolates (RSA11 and RSA42) belonging to this genus were isolated from one library and assigned to same species *Exiguobacterium indicum* on the basis of 16S rRNA gene sequences. While ANI value amongst these two strains is 94.33%, very close to the cut-off for species delineation, suggesting that these two strains may belong to two different species.

#### Genus: *Staphylococcus*

*Staphylococcus* is also a gram-positive Firmicutes and 15 different strains were selected for sequencing from this genus belonging to four different libraries and three different year rice production lots. 16S rRNA sequences have assigned them to four different species *Staphylococcus epidermidis* (SA6, SA8, SB7b, SB7c), *Staphylococcus warneri* (SA9, SA10, NS346), *Staphylococcus xylosus* (NS341), *and Staphylococcus sciuri* (NS1, NS36, NS44, NS53, NS112, NS202, RSA37). Estimated ANI values also support the species distinction between the four groups as they are above the cut-off for species delineation.

#### Genus: *Paenibacillus*

*Paenibacillus* is the bacterium belonging to gram-variable Firmicutes. One strain NS115 belonging to *Paenibacillus jamilae* was isolated from rice seed environment.

#### Genus: *Aureimonas*

*Aureimonas* is a gram-negative bacterium belonging to α-Proteobacteria. Two strains belonging to species *Aureimonas ureilytica* (NS226, NS365) were isolated from the rice seeds in two different preparations and they have ANI value of 96.91%.

#### Genus: *Methylobacterium*

Another gram-negative bacterium belonging to α-Proteobacteria, *Methylobacterium* was also isolated from rice seed environment. Five isolates from two different year rice lots belonging to two different species were extracted, *Methylobacterium radiotolerans* (SB2, SB3), and *Methylobacterium aquaticum* (NS228, NS229, NS230). ANI values also confirmed the delineation of two species.

#### Genus: *Novosphingobium*

One bacterial isolate belonging to *Novosphingobium barchaimii* NS277 was isolated that is also a gram negative α-Proteobacteria.

#### Genus: *Sphingomonas*

*Sphingomonas* is another gram-negative α-Proteobacteria. Five isolates belonging to three different species *Sphingomonas sanguinis* (SB4, NS258, and NS319), *Sphingomonas endophytica* (NS334) and *Sphingomonas yabuuchiae* (NS355) were identified using 16S rRNA gene sequences. ANI values further confirmed the species delineation.

#### Genus: *Pseudacidovorax*

*Pseudacidovorax* is a gram-negative β-Proteobacteria. One isolate belonging to *Pseudacidovorax intermedius* NS331 was isolated from rice seeds.

#### Genus: *Enterobacter*

*Enterobacter* is a gram-negative bacteria belonging to family Enterobacteriaceae of γ-Proteobacteria. Bacterial isolates belonging to this genus were isolated from three different libraries and two rice lots. Eighteen isolates were sequenced belonging to three different species on the basis of 16S rRNA sequence i.e. *Enterobacter asburia* (NS7, NS23, and NS34), *Enterobacter xiangfangensis* (NS19, NS24, NS28, NS29, NS49, NS57, NS64, NS75, NS80, NS371, RSA8), and *Enterobacter cancerogenus* (NS31, NS104, NS111, NS188). ANI values amongst all *Enterobacter asburia* and *E. xiangfangensis* strains is more than 99.9%, suggesting that isolates actually belong to one species only while their ANI value with *E. cancerogenus* strains is around 86%.

#### Genus: *Pantoea*

*Pantoea* is also a gram-negative bacteria belonging to family Enterobacteriaceae of γ-Proteobacteria. *Pantoea* strains were isolated from four different libraries from all rice lots. Seventeen different bacterial isolates were sequenced belonging to this genus with three different species *Pantoea dispersa* (SA2, SA3, SA4, SA5, NS215, NS375, NS380, NS389, RSA31), *Pantoea stewartii* (NS381, RSA13, RSA30, RSA36), and *Pantoea ananatis* (NS296, NS303, NS311, and RSA47). These isolates grouping to different species were also supported by genome based taxonomy method ANI.

#### Genus: *Pseudomonas*

The gram-negative γ-Proteobacteria *Pseudomonas* was isolated from each year rice lot and each library preparation. Fifteen isolates were sequenced that were assigned to two different species on the basis of 16S rRNA gene sequences, *Pseudomonas psychrotolerans* (SB5, SB8, SB9, SB11, SB14, SB18, NS2, NS201, NS274, NS337, NS376, NS383, RSA46), and *Pseudomonas parafulva* (NS96, NS212). While ANI analysis suggests the presence of three different species as NS337 has ANI values less than 88.5% with all other isolates.

## Author contributions

SM, KB, and VC carried out the bacterial isolations from rice seeds and their identification using 16S rRNA gene sequences. DNA isolation, QC and sequence assembly was performed by KB and SS. SM, KB, and SS did the library preparation for high throughput sequencing, run analysis and analyzed strains for genome based taxonomy. NK and PPP carried out the revival of strains for sequencing and helped in sequin file preparation. PBP conceived the study and participated in its design and coordination.

### Conflict of interest statement

The authors declare that the research was conducted in the absence of any commercial or financial relationships that could be construed as a potential conflict of interest.
